# Impact of Acetate in Reduction of Perchlorate by Mixed Microbial Culture under the Influence of Nitrate and Sulfate

**DOI:** 10.3390/ijms231810608

**Published:** 2022-09-13

**Authors:** Hosung Yu, Kang Hoon Lee, Jae-Woo Park

**Affiliations:** 1 Department of Civil and Environmental Engineering, Hanyang University, 222 Wangsimni-ro, Seongdong-gu, Seoul 04763, Korea; 2 Department of Energy and Environmental Engineering, The Catholic University of Korea, 43 Jibong-ro, Bucheon-si 14662, Korea

**Keywords:** perchlorate, mixed microbial culture, bio reduction, Monod equation, hydraulic retention time (HRT)

## Abstract

The biological reduction of slow degradation contaminants such as perchlorate (ClO_4_^−^) is considered to be a promising water treatment technology. The process is based on the ability of a specific mixed microbial culture to use perchlorate as an electron acceptor in the absence of oxygen. In this study, batch experiments were conducted to investigate the effect of nitrate on perchlorate reduction, the kinetic parameters of the Monod equation and the optimal ratio of acetate to perchlorate for the perchlorate reducing bacterial consortium. The results of this study suggest that acclimated microbial cultures can be applied to treat wastewater containing high concentrations of perchlorate. Reactor experiments were carried out with different hydraulic retention times (HRTs) to determine the optimal operating conditions. A fixed optimal HRT and the effect of nitrate on perchlorate reduction were investigated with various concentrations of the electron donor. The results showed that perchlorate reduction occurred after nitrate removal. Moreover, the presence of sulfate in wastewater had no effect on the perchlorate reduction. However, it had little effect on biomass concentration in the presence of nitrate during exposure to a mixed microbial culture, considering the nitrate as the inhibitor of perchlorate reduction by reducing the degradation rate. The batch scale experiment results illustrated that for efficient operation of perchlorate reduction, the optimal acetate to perchlorate ratio of 1.4:1.0 would be enough. Moreover, these experiments found the following results: the kinetic parameters equivalent to Y = 0.281 mg biomass/mg perchlorate, *K_s_* = 37.619 mg/L and q_max_ = 0.042 mg perchlorate/mg biomass/h. In addition, anoxic–aerobic experimental reactor results verify the optimal HRT of 6 h for continuous application. Furthermore, it also illustrated that using 600 mg/L of acetate as a carbon source is responsible for 100% of nitrate reduction with less than 50% of the perchlorate reduction, whereas at 1000 mg/L acetate, approximately 100% reduction was recorded.

## 1. Introduction

Widespread usage of perchlorate (ClO_4_^−^) in propellants, polytechnic devices and explosives for the defense production industry led to its release into the environment [1,2,3,4,5]. In addition, perchlorate has also been used in electropolishing, air bag initiators, paints, perchloric acid production and utilization, and fireworks, which contribute to further pollution while being released into the environment, particularly in water. Moreover, perchlorate also exists as a salt with the combination of various cations, including ammonium, sodium, and potassium perchlorate [6,7,8]. From these sources, 90% production of perchlorate is in the form of ammonium perchlorate due to low-cost manufacturing and ease of utilization [9,10]. Perchlorate contamination in food and water is long-lasting and toxic to humans because perchlorate requires high activation energy for degradation/reduction [1,6,11,12]. Perchlorate interferes with iodine uptake in the thyroid gland [3,13,14]. Various technologies described in the literature have been used for the reduction of perchlorate from the water and soil, which includes adsorption [15,16,17,18], membrane filtration [3,8,19,20,21,22], electrochemical reduction [10,14,23,24], biochemical reduction [1,12,25,26,27], ion exchange [11,13,28,29,30] and bio reduction [2,6,31,32,33,34]. In addition, the utilization of integrating methodologies for perchlorate reduction were also mentioned in the literature and have shown fascinating results in perchlorate reduction, which includes combination of ion exchange with bio reduction [3,11,35], but no method has provided a clear solution for the complete reduction of perchlorate.

The biological removal of perchlorate is assumed to be a promising method for the reduction of perchlorate. In previous studies, several microbial strains were used and developed for the reduction of perchlorate, which is similar to other contaminants such as nitrates, phosphate, chlorate and 1,4-dioxane [2,4,10,31,36,37,38,39,40,41]. In most of the cases, dissimilar perchlorate reducing bacteria were grown that were capable of nitrate reduction in the environment, and they preferentially utilized nitrate over perchlorate as a terminal electron acceptor, which hindered the reduction of perchlorate [6]. The bacterial strains used as the perchlorate reducing bacteria (PRBs) are *Dechloromonas* and *Dechlorsomna* (efficient for perchlorate and chlorate reduction) [31,34], *Dechloromonas PC1* and *Dechlorosomna* sp. (reduces both perchlorate and chlorate along with a transient accumulation of chlorate for utilization in syntrophic association by PRBs) [32,42], and other strains used for perchlorate reduction are denitrifying bacteria, including *Rhodobactor capsulatus*, *Rhodobactor sphaeroides*, etc. [2,4,39]. Moreover, the PRBs were isolated from a wide variety of habitats, including soils, gold mine drainage sediments, hot springs, etc. [6,31,33,40]. However, the isolation process was not energy efficient and required a high amount of energy, thus to overcome it, various acetate-oxidizing PRBs have been cultivated in which the number of bacterial cells ranged increases from 2.31 ± 1.33 × 10^3^ to 2.4 ± 1.74 × 10^6^ cells g^−1^ in a sample with same energy consumption and comparatively higher perchlorate reduction efficiency [6,43,44].

Using *Acinetobactor berziniae* Gram-negative strains as PRBs is responsible for increase in reduction to 1.33-fold by using 1.067 mM/L of anthraquinone-1-sulfonate (α-AQS) as a mediator [41]. In addition, results from other studies elucidated that washed cells (*Dechloromonas* and *Azospira* species) readily reduced 90 mg/L perchlorate in a bioelectrical reactor in the presence of a mediator (2,6-anthraquinone disulfonate (AQDS)) [10]. The cultivated microbial strains have several environmental limitations, which reduce their real wastewater application. In order to overcome the issues, mixed microbial culture was used to degrade the slow degradation contaminants, including perchlorate [5,45,46]. The mixed microbial culture provides an efficient and stable solution for perchlorate reduction by using different carbon sources as the electron donor during the process [33,45,47]. Moreover, limited studies were available on utilizing acetate as the sole carbon source for mixed microbial culture acclimation and for evaluating the impact of nitrate and sulfate on perchlorate reduction.

The goal of the study was to evaluate the impact of enriched mixed microbial culture grown using sludge from a wastewater treatment plant and using acetate as the carbon source. Moreover, the effect of nitrate on perchlorate reduction and in acclimation of mixed microbial culture was also tested along with acetate, using it as the substrate in batch testing. Moreover, the Monod equation was used to model the kinetic parameters for the perchlorate degradation by varying the acetate concentration. In addition, the variation in biomass concentration was also determined through standard methods. Loss of a carbon source in terms of COD removal was recorded throughout the study, and the impact of nitrate and sulfate as the inhibition role was also elaborated. A lab-scale aerobic reactor was used to optimize the HRT and acetate perchlorate ratio for optimum operation. 

## 2. Results and Discussions

### 2.1. Impact of Medium Enrichment by Nitrate on Perchlorate Reduction

**Figure 1:**
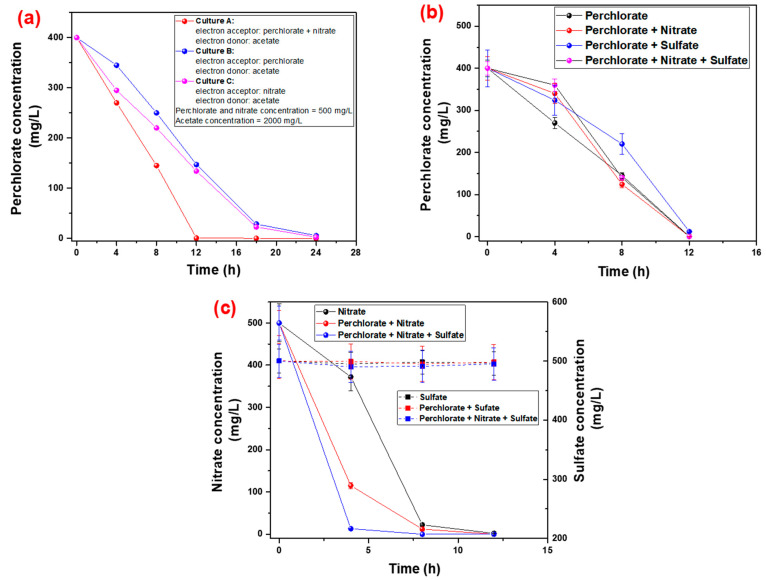
(**a**): Perchlorate reduction by mixed microbial culture grown under different combinations of perchlorate and nitrate as the electron acceptor and the acetate as electron donor; (**b**): impact of different combined electron acceptors in perchlorate reduction by culture A; (**c**): reduction of nitrate and sulfate in exposure to mixed microbial culture A (error bar represents the SD of 3 readings with the maximum SD: 8.6%) (concentration of 400 mg/L, 500 mg/L and 500 mg/L of perchlorate, nitrate and sulfate was used in feed solution as electron acceptor).

Batch testing during enrichment of mixed microbial culture with nitrate as the electron acceptor has significant effects on perchlorate reduction, as elucidated in Figure 1. As Figure 1a illustrated, culture A (nitrate and perchlorate as the electron acceptor) was responsible for the reduction of perchlorate to ≈0 mg/L after 12 h of exposure, but the mixed microbial culture B (perchlorate as the electron acceptor) and culture C (nitrate as the electron acceptor) required 24 h for the complete reduction of the perchlorate from the feed water. In addition, the inhibition impact of the different electron acceptors was also tested for the perchlorate reduction for mixed microbial culture A where perchlorate, nitrate and sulfate were used as the electron acceptor in different combinations, as shown in Figure 1b. The concentration used for the degradation analysis of perchlorate, nitrate and sulfate by culture A was 400 mg/L, 500 mg/L and 500 mg/L, respectively. The results revealed that after 12 h of exposure in batch testing, a 100% reduction in perchlorate was recorded in different combinations. In the initial stages, due to the presence of nitrate and sulfate, the reduction rate of perchlorate was slow as compared to reduction alone. However, after a significant reduction in nitrate during the initial 6 h, there was an increase in the reduction of perchlorate, which clarified that nitrate could degrade faster than perchlorate, and sulfate has little effect on the degradation of perchlorate, as shown in Figure 1c. Thus, the presence of nitrate hindered the mixed microbial culture from degrading the perchlorate due to the higher reduction affinity of nitrate. Moreover, nitrate is a faster degradation compound compared to perchlorate, and for efficient perchlorate reduction by the mixed microbial culture, nitrate concentration would be minimized [6,10,48,49].

**Figure 2:**
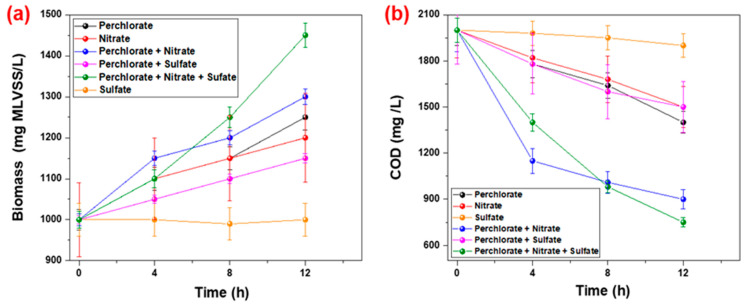
Biomass and COD variation under the influence of various electron acceptors. (**a**): Biomass concentration (mg MLVSS/L); (**b**): COD variation (mg/L) (error bar represents the SD with a reading of 3 with maximum SD: 5%).

Figure 2a illustrates the increase in biomass concentration during the reduction of perchlorate by mixed microbial culture A in the presence of different electron acceptors. The increase in biomass concentration from 1000 mg MLVSS/L to 1250 ± 31.25, 1200 ± 9.6, 1300 ± 19.5, 1150 ± 10.34 and 1450 ± 29 mg MLVSS/L were recorded for the feed solution containing perchlorate, nitrate, perchlorate + nitrate, perchlorate + sulfate and perchlorate + nitrate + sulfate solution, respectively, as the electron acceptor. The experiment was performed with a combination of nitrate + perchlorate, perchlorate + sulfate, nitrate, perchlorate, sulfate, and nitrate + perchlorate + sulfate as the electron acceptor, as mentioned above. In this study, we were unable to perform experiments in combination with nitrate + sulfate as the electron acceptor due to a major concern relating to evaluating the perchlorate reduction. Moreover, the degradation rate of nitrate is faster than perchlorate, and perchlorate degradation starts after the complete degradation of nitrate, so we expected that the presence of sulfate facilitated the nitrate reduction due to biomass production. However, individually it would have no significant effect. Furthermore, faster degradation of perchlorate and nitrate by a mixed microbial culture provides proof of the compatibility of these two chemicals in the microbial community, which was ultimately responsible for the reduction of the feed COD. The acetate concentration was recorded as the COD during the process and elucidated in Figure 2b. The reduction in the COD was from 2000 mg/L to 1400 ± 70, 1500 ± 119, 900 ± 63, 1500 ± 124 and 750 ± 30 mg/L for perchlorate, nitrate, perchlorate + nitrate, perchlorate + sulfate and perchlorate + nitrate + sulfate solution, respectively. The reduction in the COD was particularly high in the feed containing the perchlorate combination with nitrate and nitrate + sulfate. Due to the faster reduction of nitrate as compared to the perchlorate, it was elucidated that after the nitrate reduction, the microbial culture reduction potential for the perchlorate was enhanced due to a rapid increase in the biomass concentration (due to the presence of an active microbial community). However, no significant variation in a reduction in sulfate was observed in a mixed microbial culture where acetate was the primary source of the electron donor; thus, no variation in the COD and biomass concentrations of sulfate as the electron acceptor during 12 h of exposure time were observed.

### 2.2. Optimizing the Electron Donor Dose for Perchlorate Reduction

**Figure 3:**
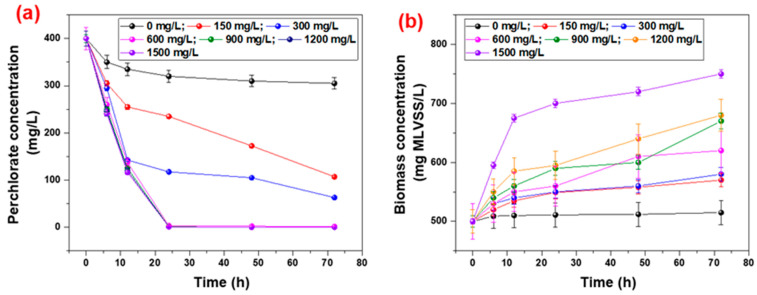
Impact of acetate concentration on perchlorate reduction by a mixed microbial culture and biomass concentration (**a**): perchlorate reduction (mg/L); (**b**): biomass concentration (mg MLVSS/L) (error bar represents the SD with a reading of 3 with maximum SD: 5%).

A batch test reactor was used to evaluate the endogenous decay rate of perchlorate at different concentrations of acetate as an electron donor component. The fixed concentration of 400 mg/L of perchlorate for it and the results are shown in Figure 3a. The endogenous decay of perchlorate by enriched microbial culture under a dose of acetate concentration from 0 to 1500 mg/L for 72 h revealed that the reduction in perchlorate increased with an increase in the concentration of acetate. The results showed that after 72 h, the concentration of 305.17 ± 12.2, 107.24 ± 2.14, 63.7 ± 1.26 and less than 1.5 ± 0.09 mg/L were observed for acetate concentrations of 0, 150, 300 and greater than 600 mg/L, respectively. Moreover, we considered the concentration of 600 mg/L to be optimum, as no significant difference was recorded for a decrease in perchlorate concentration. Figure 3a also shows that 24 h was enough for the retention time of the maximum degradation of perchlorate at 600 mg acetate/L. The figure also defined the optimum acetate to perchlorate ratio for the complete removal of perchlorate, which was 1.4 mg acetate/mg perchlorate at an acetate concentration of 600 mg/L.

An initial biomass concentration of 500 mg MLVSS/L was used for the batch experiment for endogenous decay of the perchlorate, and the results are shown in Figure 3b. The results show that with an increase in acetate concentration, the production of biomass increases due to the utilization of acetate and perchlorate as food by an enriched mixed microbial culture and significantly decreases the concentration of perchlorate and acetate in the form of COD. The increase in biomass from 500 mg MLVSS/L to 515.65–750.79 mg MLVSS/L was observed for 0–1500 mg/L of acetate at 400 mg/L of perchlorate. Specific substrate utilization was recorded at the 600 mg/L of acetate concentration to evaluate the kinetic coefficients of the degradation of perchlorate for 24 h experiments, and the results are summarized in Table 1. The results showed a good correlation with the previous studies, in which mixed microbial culture was used for the perchlorate reduction with different food sources [5,49]. Logan et al. [45] used the fixed bed bioreactor along with ethanol for the perchlorate reduction and elucidated the optimized kinetic parameters (*K_s_* = 20 mg/L; q_max_ = 1.821 mg/mg·d) for the reduction in perchlorate in the drinking water level. Moreover, the reaction kinetics of perchlorate degradation showed that 190 mg/L of sodium acetate as a carbon source would be enough for complete degradation in the absence of any inhibition chemicals (such as nitrate) [33,45]. Similar results were found in which a single strain of microbial culture was used for the degradation of perchlorate [49]. Yu et al. [49] explained the reduction of perchlorate by using the combined PRMs and zero-valent ions in batch testing where kinetic parameters of *K_s_* = 8.9 mg/L; q_max_ = 0.65 mg/mg.d were determined and responsible for the increase in perchlorate reduction of up to 4 times compared to a single process. Furthermore, the addition of nitrate and pH delays the reduction in perchlorate, but it is not inhibited completely. 

### 2.3. Lab Scale Testing

**Figure 4:**
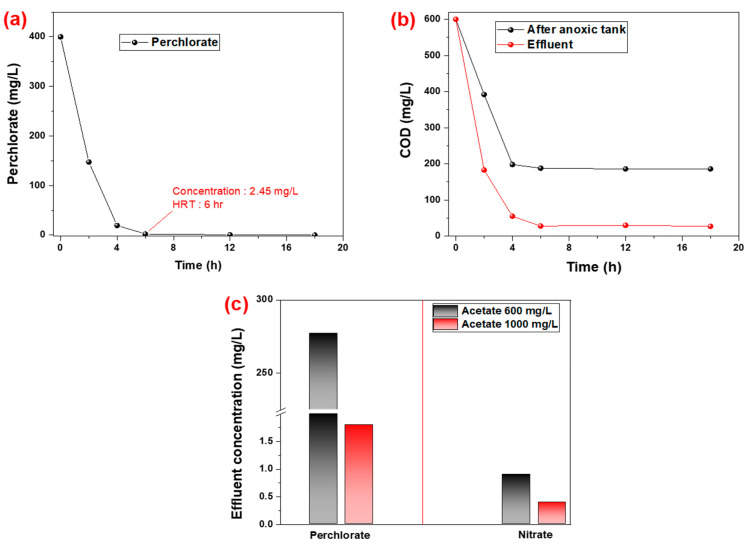
Lab scale operation of activated sludge treatment for perchlorate biodegradation by mixed microbial culture. (**a**): HRT optimization at a biomass concentration of 1000 mg MLVSS/L; (**b**): COD variation from the reactor while optimizing the HRT; (**c**): impact of acetate concentration on perchlorate and nitrate reduction at 6 h HRT.

Lab scale experiments were conducted at different HRT times ranging from 0 to 18 h for five days, each with a 48 h adaption time of the reactor. The results on perchlorate degradation and COD variation are given in Figure 4. The results show that with an increase in the HRT, the degradation of perchlorate increased, as shown in Figure 4a. We considered an HRT of 6 h as optimum because most of the perchlorate was degraded, and the concentration was 2.45 mg/L. In addition to the reduction of perchlorate, the carbon source (acetate) also decreased to a level of 28 mg/L in terms of the COD, and no further reduction was observed for greater than 6 h HRT as seen in Figure 4b. The COD concentration results for the anoxic tank showed that a major reduction took place in the anoxic tank and that recycling of the active biomass played a significant role in the faster degradation of acetate and perchlorate. 

Additional experiments to evaluate the impact of nitrate and the concentration of acetate were also performed, and the results showed that despite the carbon source concentration of the mixed microbial culture, the nitrate experienced a faster degradation rate compared to the perchlorate, as seen in Figure 4c. At a lower concentration of carbon source, there was a 100% nitrate reduction, but perchlorate had less than a 50% reduction, while at a 1000 mg/L acetate dose, both had a ≈100% reduction, which strengthened the observation of the inhibition potential of nitrate toward the perchlorate and was responsible for slowing down its degradation. Thus, for efficient reduction of perchlorate through feed water by a mixed microbial culture, it is highly recommended to remove the nitrate from the feed water or to provide additional carbon, which should be higher than a 1.4 mg acetate/mg perchlorate ratio. Moreover, the pilot scale plant associated with the combination of a plug flow reactor was planned for the real wastewater application for perchlorate degradation through a mixed microbial culture at these design parameters.

## 3. Materials and Methods

### 3.1. Materials

**Figure 5:**
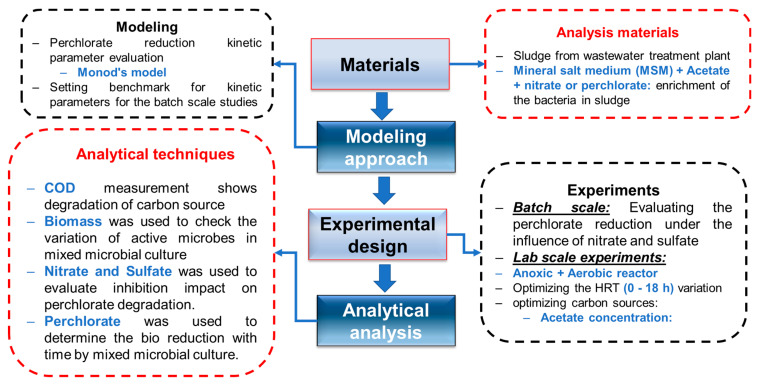
Schematic diagram of the experimental study.

Anaerobic sludge was collected from the digester of a wastewater treatment plant in Suwon, South Korea and used as the seed inoculum for the enrichment of the perchlorate-reducing culture. A mineral salt medium (MSM) was used to grow the mixed culture and enrichment prepared in ultra-pure water according to the methodology mentioned elsewhere [36,38]. The chemicals for MSM preparation were of analytical grade. Concentration and chemicals included in MSM were NH_4_Cl (2000 g/L), KH_2_PO_4_ (1000 g/L), Na_2_HPO_4_·2H_2_O (1000 g/L), MgSO_4_·7H_2_O (100 g/L), FeSO_4_·7H_2_O (12.6 g/L), NaMoO_4_·2H_2_O (10 g/L), H_3_BO_3_ (0.6 g/L) and NaSeO_3_ (0.6 g/L). The sodium perchlorate solution was obtained from Sigma-Aldrich Chemicals, Darmstadt, Germany. Figure 5 shows an overview of the experimental study for the bioreduction of ClO_4_^−^.

### 3.2. Enrichment of Perchlorate Reducing Mixed Bacteria

Enrichments, growth of mixed culture and all experiments were performed in an MSM with the predetermined concentration of perchlorate as the electron acceptor and acetate as the electron donor. They were injected into prepared synthetic wastewater and placed in a bottle for stirring. Synthetic wastewater was prepared by dissolving the predetermined mass of pollutants in DI water, in our case, perchlorate with the combination of nitrate and sulfate. After culturing for 24~48 h, the microorganisms were centrifuged and injected again into newly prepared synthetic wastewater. The anaerobic sludge was centrifuged, all the supernatant was decanted and the settled sludge was washed with MSM two to three times. The sludge obtained after washing was used for the experiment. 1000 mg/L acetate and 500 mg/L perchlorate were added to four 500 mL bottles, each containing equal amounts of biomass and 300 mL of the MSM. All the bottles were capped and shaken at 150 rpm at room temperature (21 ± 2 °C). After 48 h, the biomass was allowed to settle for 1 h. The supernatant was decanted and replaced with a fresh MSM, along with the addition of 1000 mg/L of acetate and 500 mg/L of perchlorate before the start of the next cycle.

### 3.3. Experimental Procedure and Reactor Design

The batch scale experiments were conducted with a fixed biomass concentration of 1000 mg MLVSS/L to evaluate the bioreduction of perchlorate. Moreover, the effect of the medium (enriched mixed culture) acclimation with or without nitrate on perchlorate reduction was also examined. For that, we used 500 mL bottles containing three different acclimated cultures, containing perchlorate (500 mg/L), nitrate (500 mg/L) and acetate (2000 mg/L) in different combinations: ***Culture A*** (perchlorate and nitrate as the electron acceptor, acetate as the electron donor), ***Culture B*** (perchlorate/acetate as the electron acceptor/donor) and ***Culture C*** (nitrate/acetate as the electron acceptor/donor).

We also examined the effect of acetate concentration to determine the optimum relationship between acetate concentration and perchlorate by using the acetate concentration range of 0–1500 mg/L, which was used for medium acclimation to evaluate the reduction of a fixed 400 mg/L perchlorate concentration. In this experiment, 300 mL of mixed microbial culture amended with an acetate concentration of 0–1500 mg/L was shifted to different 500 mL bottles, which contained fixed 400 mg/L perchlorate. The bottles were purged with N_2_ and then sealed and incubated in a shaker at 150 rpm. All the experiments were conducted at room temperature (21 ± 2), and the samples were collected at regular intervals for analysis. Moreover, these batch scale experiments were conducted to evaluate the decaying parameters by Monod’s equation, as mentioned in Section 3.5. Modeling Approach.

**Figure 6:**
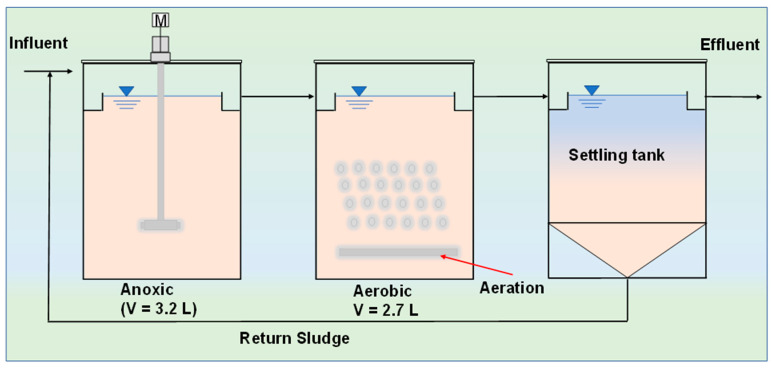
Schematic of lab scale reactor setup with a schematic for biodegradation of perchlorate.

A continuous reactor was used to determine the optimized hydraulic retention time (HRT) for the bioreduction of perchlorate under the predetermined optimized acetate and nitrate concentration; the schematic is shown in Figure 6, which shows the anoxic tank and aerobic tank of 3.2 L and 2.7 L. The pump was used to regulate the flow and HRT of the system. The anoxic tank was used to maintain the mixed liquid volatile suspended solids (MLVSS) in suspension by a stirrer. Moreover, a small amount of the mixed microbial culture was injected with 400 mg/L of perchlorate for 48 h in the anoxic tank as an adaptation time. Consideration of 48 h as the adaptation time was based on the batch test analysis. In the batch test, 24 h was used for the degradation of perchlorate in 500 mL bottles by various enriched mixed microbial cultures; thus, we expected that in a larger tank, the bacteria need more time to adapt to the environmental conditions. Therefore, we chose the 48 h adaptation time in an anoxic tank. Air was supplied to the aerobic tank from the bottom at a uniform rate, and sludge was returned from the settling tank to the anoxic tank. The samples were collected after every 24 h period for five days of operation for each HRT duration. The perchlorate concentration, chemical oxidation demand (COD) and nitrate concentration were measured (the average results are given). The biomass concentration of 1000 mg MLVSS/L was maintained during the process. 

### 3.4. Analytical Procedure

Aliquot samples of 2 mL from bottle tests were taken by using a 5 mL sampling syringe, filtered through a 0.2 μm membrane filter and stored in a refrigerator at 4 °C prior to being analyzed. Perchlorate was measured using Liquid Chromatograph/Mass Spectrometry (LC/MS) equipped with an XBrige^TM^ C18 3.5 µm, 2.1 mm × 100 mm column, Waters, Milford, MA, USA and a conductivity detector. The detection limit for perchlorate was 0.05 mg/L. 0.1% formic acid in distilled water, 0.1% formic acid in methanol, 10% methanol and 90% methanol served as the eluent. The flow rate of the eluent was 0.4 mL/min. A 100 mg/L of perchlorate solution was confected to prepare perchlorate calibration standards. Moreover, the COD was measured for acetate concentration measurement and measured by centrifuging the liquid culture sample at 14,000 rpm for 5 min to separate the biomass portion completely. An appropriate volume of supernatant was used for COD measurement in accordance with the standard method.

The biomass concentration was measured by a mass measuring method. An appropriate 50 mL volume of culture was filtered (vacuum filtration by using 0.2 μm PTFE standard filter paper) and weighed after the samples were dried at 105 °C for 4 h, which gave the biomass concentration value. Nitrate was measured using the Chromotropic Acid Method; the high range (0.2 to 30.0 mg/L NO_3_--N) and sulfate were measured using the SulfaVer 4 Method (2 to 70 mg/L SO_4_^2−^) with HACH spectrophotometry at 410 nm and 450 nm wavelength.

### 3.5. Modeling Approach

We investigated the biodegradation of ClO_4_^−^ by various microbial metabolism processes of pure cultures, with a single strain isolated and characterized for ClO_4_^−^ biodegradation. Generally, the perchlorate-reducing bacteria derived from the “16S rDNA sequence” belongs to the subclasses of *Proteobacteria* (α, β, γ and ε), as mentioned earlier [6,33,41]. However, in this study, the mixed culture for the perchlorate degradation was tested by an anoxic–aerobic bioreactor for the lab scale, which was limited in previous studies, as shown in Figure 6. The coefficient of kinetic parameters for these reactors for the rate of perchlorate utilization was determined by applying the Monod model Equation (1).

(1) $$\frac{dS}{dt}=-\frac{q_{m}S}{K_{s}+S}\left[X_{a}^{0}+Y_{t}\left(S_{0}-S\right)\right]$$

where *K_s_* represents half-saturation coefficients (mg ClO_4_^−^/L), *q_m_* represents the maximum specific substrate utilization ((mg ClO_4_^−^/mg-MLSS/h), *Y_t_* represents the true cell yield (mg-MLSS/mg ClO_4_^−^), *X_a_*^0^ represents the concentration of the biomass initially, (mg/L) *S*_0_ represents the concentration of the substrate initially (mg/L) and *S* represents the concentration of substrate at a function of time *t* (mg/L), as mentioned earlier [9,45,49]. The batch scale experiments were conducted to determine the substrate utilization constants and estimate the unknown parameters, where the optimized concentration of acetate was used from a range varying from 0 to 1500 mg/L as the electron donor in Equation (1).

The observed yield strength of the cell growth was estimated by the linearization of the substrate (1,4-Dioxane) utilization, as shown in Equation (2).

(2) $$Y_{ob}=\frac{dX_{a}/dt}{dS/dt}=\frac{C_{growth}}{Perchlorate_{utilization}}$$

## 4. Conclusions

All mixed microbial cultures acclimated with and without nitrate had the ability to reduce perchlorate in anaerobic conditions. Moreover, negligible reduction in sulfate concentration was observed by mixed culture and had no significant contribution to perchlorate reduction. However, there was an increase in biomass production in the presence of all three chemicals as electron acceptors, which tended to reduce the COD level and increase the perchlorate degradation. Additionally, we confirmed that prior to perchlorate degradation, nitrate removal took place, which showed that an enriched mixed microbial culture acclimated with acetate as a carbon source has a great affinity for nitrate and was an inhibition factor for the perchlorate. With an excess carbon source, the effect of various concentrations of nitrate on perchlorate reduction was similar. In the batch experiment, the optimal acetate to perchlorate ratio of 1.4 mg acetate/mg perchlorate was observed at 600 mg/L of acetate concentration. Along with that the kinetic parameters of the Monod equation were Y = 0.281 mg biomass/ mg perchlorate, *K_s_* = 37.619 mg/L and q_max_ = 0.042 mg perchlorate/ mg biomass/h. The reactor experiment to determine the optimal operation condition had an HRT of 6 h with 600 mg/L of acetate. However, in elucidating the impact of nitrate at optimal conditions, a reduction in perchlorate was observed with a removal efficiency of less than 60% and complete removal of nitrate due to the inhibition impact of nitrate. Furthermore, an increase in the carbon source to 1000 mg/L achieved the 100% removal of nitrate and perchlorate at 6 h HRT time. These results illustrated the significance of mixed microbial culture for the removal of perchlorate in an anaerobic environment and that it is necessary to reduce the nitrate concentration prior to perchlorate reduction or to provide an additional carbon source for simultaneous degradation of nitrate and perchlorate. Furthermore, through a reactor experiment, we also verified that the acetate concentration as a carbon source has a significant impact on the reduction of nitrate and perchlorate. Future experiments on designing real-world applications for wastewater treatment plants are under consideration for a continuous flow reactor with various contaminants of inhibition.

## Figures and Tables

**Table 1:** Summary of kinetic parameter evaluation by a batch test of perchlorate biodegradation.

Kinetic Parameters	Values
*K_s_* (mg Perchlorate/L)	37.61
q_max_ (mg Perchlorate/mg MLVSS/d)	0.042
*Y_t_* (mg MLVSS/mg Perchlorate)	0.281
